# Development of the college student life satisfaction scale (CSLSS): initial validation among Filipino college students

**DOI:** 10.3389/fpsyg.2025.1560997

**Published:** 2025-07-16

**Authors:** Ramon Paulo E. Masagca

**Affiliations:** ^1^Department of Psychology, Manila Central University, Caloocan, Metro Manila, Philippines; ^2^Department of Psychology, University of the Philippines – Diliman, Quezon City, Philippines

**Keywords:** student life satisfaction, measurement, school-life balance, pandemic, Filipino

## Abstract

Few domain-specific satisfaction measures explicitly evaluate students’ satisfaction with their learning experiences. Although numerous instruments are available to measure student satisfaction, they often focus on general life domains rather than specific aspects of the learning experience. Furthermore, it is imperative to ensure that these measures effectively capture learning experiences, particularly within the context of Filipino college students. The development of the College Student Life Satisfaction Scale (CSLSS) addresses the need for a culturally relevant instrument that reflects the unique learning experiences of this population. It also considers the impact of changes in higher education during the COVID-19 pandemic, emphasizing the importance of school-life balance as a key aspect of student life satisfaction. The CSLSS comprises five dimensions: Peers, Faculty, School Environment, Academic Performance, and School-Life Balance. These dimensions were validated through exploratory factor analysis using responses from *n* = 406 Filipino college students enrolled in the academic year 2024–2025. The final version of the CSLSS, validated with a sample of *n* = 327, was found to be a reliable and valid measure of student life satisfaction. It exhibits concurrent validity with the College Satisfaction Scale and the Multidimensional Student Life Satisfaction Scale, and its construct validity is demonstrated through significant correlations with the Academic Hope Scale and the Satisfaction with Life Scale. While objective assessments are essential for informing educational reform, the inclusion of students’ subjective experiences provides critical insights. The integration of both approaches supports a more comprehensive strategy for enhancing educational quality.

## Introduction

Life satisfaction, a key component of subjective wellbeing alongside positive and negative affect, is considered a cognitive evaluation of one’s life (Diener et al., 1985). While related to affective states, it differs from them (Lucas et al., 1996). A central question is how people make evaluative judgments about their lives.

Schwarz and Strack (1999) suggest that life satisfaction evaluations rely on readily available information rather than comprehensive assessments, with mood influencing judgments. Schwarz and Clore (1983) found college students reported higher life satisfaction on sunny days, while Sinclair and Mark (1995) showed that positive moods result in less accurate recall than negative moods. Question order also affects life satisfaction; Strack et al. (1988) demonstrated that asking about dating before life satisfaction led to a stronger correlation.

Despite these transient effects, life satisfaction is primarily shaped by stable, accessible information. Schimmack and Oishi (2005) showed that global life satisfaction, as measured by the Satisfaction with Life Scale, remains stable and is linked to domains like family and housing, with factors such as weather being irrelevant. Chronic health conditions also affect satisfaction, with McNamee and Mendolia (2014) finding that health issues lower life satisfaction by influencing critical domains like employment and relationships, even when controlling for income and employment status.

Lucas et al. (2018) found that single-item life satisfaction scales are stable over time, suggesting that fluctuations may reflect measurement error rather than actual changes. Life satisfaction judgments follow a bottom-up process, based on the importance and comparison of specific life domains (Lucas, 2004). Campbell et al. (1976) identified the need to assess and compare one’s condition in those domains. For example, a person might have negative life satisfaction due to a family illness, while another has positive satisfaction due to a cohesive family, regardless of external factors like weather. Therefore, life satisfaction is shaped by stable, significant domains rather than transient factors.

Young people often base their global life satisfaction on various domains, with school being a key factor. For instance, a college student in a satisfying learning environment is likely to form a positive global life judgment. This experience is influenced by factors such as quality of instruction, faculty support, student services, peer relationships, and family involvement. Investigating these factors is crucial for understanding student life satisfaction and enhancing student wellbeing. Educational institutions can improve services and fulfill their commitment to student success by investing in these areas. One way to achieve this is through the development of a reliable, contextually appropriate tool to measure student satisfaction with learning experiences.

However, many existing tools assess satisfaction across domains beyond the immediate context of higher education. For example, the Multidimensional Student Life Satisfaction Scale (MSLSS; Huebner and Gilman, 2002) includes a “school” dimension but also covers other areas not directly relevant to the academic experience. Similarly, the College Satisfaction Scale (Lodi et al., 2017) measures satisfaction with education but is specific to Italian students, raising concerns about its applicability in other cultural and educational settings. In particular, the scale may not fully capture the unique experiences of Filipino students.

Furthermore, the relevance of these existing scales must be reconsidered in light of recent shifts in educational paradigms. The COVID-19 pandemic has brought about significant changes in the modalities of higher education, especially in college settings, leading to alterations in how students engage with their learning environments (Cheung et al., 2023). Consequently, students’ evaluative judgments of their academic experiences may have evolved, with greater emphasis now placed on the balance between academic responsibilities and personal wellbeing. This shift underscores the need for an updated approach to measuring student life satisfaction—one that reflects the current landscape of higher education. Existing assessment tools may no longer fully account for these changes, necessitating the development of new measures that are better aligned with the evolving priorities of students.

### Significance of the study

This study aims to develop the “College Student Life Satisfaction Scale (CSLSS),” a tool designed to measure college students’ overall satisfaction with their student experience. The CSLSS will be the first contextual life satisfaction measure for college students, addressing aspects such as academic programs, teaching quality, campus facilities, peer relationships, and support services. The scale will also account for cultural influences, particularly the collectivist culture of Filipinos, which may shape students’ perceptions and priorities.

The CSLSS can be utilized by higher education institutions (HEIs) in regular student feedback surveys to assess satisfaction. It will help identify strengths and weaknesses in areas like teaching methods or campus facilities, informing targeted improvements. By capturing the multifaceted nature of student life satisfaction, a valid tool will highlight areas needing attention to improve student wellbeing. HEIs can use this information to implement programs focused on enhancing these domains. Additionally, studies on student life satisfaction can explore topics such as the relationship between academic services and overall satisfaction, the impact of extracurricular involvement on wellbeing, and satisfaction comparisons across demographic groups.

### Rationale of the study

Student life satisfaction is influenced by how well students’ needs are met in the academic environment. The COVID-19 pandemic disrupted these needs, particularly with the shift to remote learning, which diminished school-life balance (SLB) as the separation between school and personal life blurred. This mirrors boundaryless work environments, where “anytime-anywhere” accessibility impedes detachment and negatively affects life outside of work (Park et al., 2011; Mellner, 2016). Health sciences students also reported declines in wellbeing during the early pandemic (Pérez-Villalobos et al., 2023), highlighting the need to address SLB and other critical needs in higher education.

Identifying school-related domains most associated with student life satisfaction is crucial for educational institutions. While tools like the MSLSS and BMLSS are commonly used, concerns about their contextual relevance and psychometric properties persist. To provide a more accurate and contextually appropriate measure of college students’ life satisfaction, especially related to their learning experiences, the development of the College Student Life Satisfaction Scale has been proposed.

## Review of related literature

### Higher education institutions in the Philippines during the COVID-19 pandemic

The COVID-19 pandemic significantly impacted higher education institutions (HEIs) in the Philippines. In a study conducted by Miranda and Tolentino (2023), nearly half of the 3,718 student respondents reported experiencing mild to extremely severe levels of depression, anxiety, and stress. Notably, students in lower year levels were found to be more vulnerable to heightened psychological distress. This strain was likely exacerbated by intensified academic demands and abrupt shifts in learning modalities. Supporting this, Berdida and Grande (2023) emphasized that academic stress—compounded by pandemic-related anxiety—had a detrimental effect on students’ overall wellbeing (Pérez-Villalobos et al., 2023; Berdida and Grande, 2023).

One of the most affected dimensions of student wellbeing during the pandemic was school-life balance. The Commission on Higher Education (CHED, 2020) mandated a shift to online learning for HEIs to ensure the continuity of education while complying with public health protocols. This abrupt transition required students to transform their homes—or even their bedrooms—into full-time learning spaces. Consequently, many students struggled to maintain clear boundaries between academic responsibilities and personal life, making it difficult to manage roles and routines. This blurred separation between domains adversely impacted students’ overall wellbeing (Park et al., 2011; Mellner, 2016; Gao et al., 2022).

The Networked Learning Editorial Collective (NLEC et al., 2021) further noted that the absence of physical separation between study and personal environments altered students’ perceptions of their educational experiences. Many described the learning process as invasive and less structured due to the erosion of spatial boundaries. This condition led to emotional and psychological spillover, as students reported difficulty “switching off” from academic demands—resulting in fatigue, anxiety, and a growing sense of isolation.

At present, higher education institutions in the Philippines continue to recover from the disruptions brought about by the pandemic. However, the experience has yielded valuable insights. One key realization is that online learning modalities can serve as viable alternatives to in-person instruction, particularly during class suspensions caused by inclement weather or health-related concerns. This shift highlights a broader recognition of the importance of flexible and adaptive learning systems—not only as logistical solutions but also as responses to students’ evolving academic and psychological needs.

### Satisfaction of basic psychological needs in the academia

Self-Determination Theory (SDT; Deci et al., 2001) posits that the satisfaction of three basic psychological needs—autonomy (the need to feel in control of one’s actions), competence (the need to feel effective in one’s activities), and relatedness (the need to feel connected to others)—leads to optimal psychological growth, integrity, and wellbeing. Numerous studies across various cultures and life domains have consistently supported this theoretical claim.

For instance, Ryan and Deci (2001) synthesized findings from education, work, and clinical settings, concluding that individuals who experience greater satisfaction of autonomy, competence, and relatedness report higher levels of life satisfaction, intrinsic motivation, and psychological wellbeing. In the educational context, Deci et al. (1991) found that students in autonomy-supportive classrooms exhibited higher intrinsic motivation, greater self-esteem, and stronger academic engagement compared to those in more controlling environments. In the healthcare domain, Ng et al. (2012) conducted a meta-analysis of 184 studies involving over 61,000 participants across 10 countries and found that need satisfaction consistently predicted improved health behaviors—such as medication adherence and physical activity—as well as enhanced psychological health. In the prosocial and interpersonal domain, Weinstein and Ryan (2010) demonstrated that individuals who engaged in helping behaviors out of autonomous motivation (rather than obligation) experienced greater vitality and lower stress, both for themselves and for those they helped.

In the context of this study, academia serves as a key context through which students can satisfy their basic psychological needs. Meaningful interactions with peers and professors, along with the autonomy to choose an academic program or track, provide students opportunities to fulfill their needs for relatedness and autonomy. Furthermore, the institution plays a critical role in fostering students’ sense of competence by offering academic challenges, accessible resources, and support systems that promote skill development and mastery (Gilbert et al., 2021).

Perceived autonomy support from peers has been shown to strongly predict academic and general life satisfaction, as well as positive and negative affect, even more so than support from parents or romantic partners (Ratelle et al., 2013). As young adults transition into adulthood, peer support increasingly contributes to competence and mastery, gradually replacing the earlier role played by parents (Surjadi et al., 2011; Collins and Madsen, 2006). For example, among South African pharmacy students, peer support was found to enhance competence satisfaction (Basson and Rothmann, 2018).

Teachers also play a central role in supporting students’ psychological needs. Law students who perceived their professors as supportive reported greater satisfaction of their basic psychological needs, better academic performance, and higher motivation (Sheldon and Krieger, 2007). Conversely, controlling teaching behaviors have been associated with lower levels of need satisfaction and poorer outcomes. Teachers also help address students’ social and emotional needs and can play an important role in identifying mental health concerns (Di Placito- Rango, 2018). Instructor–student relatedness has been shown to influence academic motivation and outcomes, including students’ perceptions of learning and academic performance (Fedesco et al., 2019).

Institutional factors also contribute to psychological need satisfaction. For instance, Blended Learning Environments (BLE) show potential in this regard. Learning analytics have been used to personalize content, thereby supporting autonomy and competence, while workshops and co-curricular activities enhance competence and relatedness (Ameloot et al., 2024). Similarly, mixed-mode learning has been found to foster relatedness through improved communication and to support competence through deeper learning and online assessments (Wong, 2022). However, autonomy may be less supported in these settings due to limitations in assessment methods and prevailing school culture.

*Thwarted needs*. There is a notion that a particular life domain becomes a salient reference for an individual’s life satisfaction judgment when they have experienced deprivation or depletion in that domain. This idea is supported by Self-Determination Theory (SDT), particularly its proposition regarding the consequences of thwarted psychological needs. In their seminal work, *“The ‘What’ and ‘Why’ of Goal Pursuits: Human Needs and the Self-Determination of Behavior,”* Deci and Ryan (2000) explain how unmet needs—such as autonomy, competence, and relatedness—gain psychological salience and influence motivation and wellbeing. During the COVID-19 pandemic, students’ need for autonomy was arguably thwarted, as they had little to no choice in the modality of learning they received. In most cases, they were required to attend online or distance learning. The lack of distinction between school and personal life contributed to a diminished sense of control, especially in managing responsibilities outside of academics (e.g., family roles). As a result, students were more likely to experience “ill-being” rather than wellbeing or student life satisfaction. In more stable conditions, such as the post-pandemic period, these previously thwarted needs are expected to gain greater psychological salience—reflected in students placing increased value on maintaining school-life balance. Thus, it is expected of students to actively “satisfy” these “thwarted” needs (school-life balance) through various means. Students tend to favor flexible learning approaches that are customizable alongside other life responsibilities more after the pandemic in which online learning is not a choice but a required protocol (Dos Santos, 2022; El Galad et al., 2024; Koh and Daniel, 2022; Sato et al., 2023; Thahir et al., 2023; Van der Westhuizen and Hlatshwayo, 2023). These approaches are consistently associated with greater academic engagement and improved learning outcomes. Thus, school-life balance emerges in the present study as a significant contributor to student life satisfaction.

Overall, the satisfaction of school-related basic psychological needs—including those that were previously thwarted—enhances students’ sense of fulfillment in their academic experience. As college is widely regarded as a critical and formative stage in youth development, a positive academic environment is likely to shape broader aspects of students’ wellbeing. Consequently, college student life satisfaction functions as a vital link between the fulfillment of school-related psychological needs and students’ overall life satisfaction.

### Student life satisfaction and global life satisfaction

A significant portion of a child’s time is spent in academic institutions, making school experiences a key influence on life satisfaction. Huebner et al. (2014) identified school-related factors—teacher and peer interactions, parental involvement, academic performance, safety perceptions, instruction quality, and extracurricular opportunities—as major contributors to wellbeing and life satisfaction. Schools, alongside families, are crucial in shaping subjective wellbeing, as shown by the Brief Multidimensional Student Life Satisfaction Scale (Seligson et al., 2003), which highlights the positive impact of family, friends, school, living environment, and self-reflection on global life satisfaction.

Within schools, teacher-student relationships are particularly influential, with supportive interactions fostering positive emotions and wellbeing. Teacher relationship satisfaction often outweighs peer influence in enhancing life satisfaction (Natvig et al., 2003). High global life satisfaction is linked to better academic, interpersonal, and intrapersonal functioning, greater hope, and a stronger sense of personal control (Gilman and Huebner, 2006). Conversely, bullying negatively impacts wellbeing (Flouri and Buchanan, 2002).

Academic performance significantly correlates with life satisfaction. Students who perceive themselves as academically competent report higher life satisfaction, with this relationship remaining stable over time (Leung et al., 2004). Studies show that academic success fosters life satisfaction, while higher life satisfaction enhances academic outcomes through increased self-esteem, self-efficacy, school engagement, and problem-focused coping (Huebner and Alderman, 1993; Huebner et al., 2014; Ng et al., 2015).

School environments affect life satisfaction, with factors such as safety, fairness, and a positive climate enhancing educational experiences and wellbeing (Samdal et al., 1998). Unsafe environments or experiences like theft and violence reduce life satisfaction (Valois et al., 2001). Large-scale studies show that rigorous systems in Japan and Korea correlate with high stress and lower wellbeing, while Finland’s low-anxiety approach fosters better life satisfaction and educational outcomes (Kirkcaldy et al., 2004).

Non-academic activities also improve school satisfaction. Students engaged in extracurricular activities report higher life satisfaction, promoting academic involvement and overall wellbeing (Suldo and Huebner, 2006). Furthermore, the school experience influences post-school life, with transitioning directly from education to employment leading to higher life satisfaction due to reduced financial stress and increased career decision-making self-efficacy (Creed et al., 2003).

### Existing measures of student life satisfaction

Life satisfaction scales typically follow three models: unidimensional (global and general) and multidimensional. Global life satisfaction scales use context-free items that allow individuals to assess aspects of their lives they consider important (Pavot and Diener, 1993). General life satisfaction scales aggregate ratings across predetermined domains like relationships, health, and personal development (Gilman et al., 2000).

Multidimensional scales, such as the Multidimensional Student Life Satisfaction Scale (MSLSS; Huebner and Gilman, 2002), offer domain-specific scores for a more detailed view of life satisfaction but do not fully capture students’ satisfaction with learning experiences. For example, the MSLSS’s School domain broadly addresses school experiences but lacks specificity on factors like faculty competence and social support.

The Brief Multidimensional Students Life Satisfaction Scale (BMSLSS; Seligson et al., 2003) condenses the MSLSS into five general items, but single-item measures lack the specificity needed to identify factors influencing life satisfaction (Cheung and Lucas, 2014). The College Satisfaction Scale (CSS; Lodi et al., 2017) measures life satisfaction specific to college experiences, but it was validated only among Italian students. In contrast, the College Student Life Satisfaction Scale (CSLSS) was developed for Filipino students to address cultural and contextual differences.

Cultural factors significantly influence how individuals make judgments about life satisfaction. For instance, Jiang et al. (2021) found that the item “I have what I want in life” functioned differently among Chinese and American students, reflecting distinct cultural orientations. In Western contexts, life satisfaction is often framed in terms of personal achievement and self-actualization, whereas in collectivist cultures, it is more closely tied to interpersonal relationships and social harmony (Markus and Kitayama, 1991). Supporting this, Veronese and Pepe (2020) reported that the Self domain of the Multidimensional Student Life Satisfaction Scale (MSLSS) was not applicable to Palestinian children living in refugee camps—an outcome attributed to the collectivist values of their sociocultural environment (Giacaman, 2017). These findings collectively underscore the limitations of applying universal life satisfaction models across diverse cultural settings and affirm the need for culturally sensitive assessment tools (Markus and Kitayama, 1991; Suh et al., 1998). In the Philippine context, there remains a lack of developed or culturally adapted instruments specifically designed to assess college student life satisfaction, highlighting the relevance and necessity of localized scale development.

### Academic hope

This study establishes the convergent validity of the constructed scale by demonstrating the directional relationship between academic hope and student life satisfaction.

Bailey et al. (2007) explored the relationship between hope, optimism, and life satisfaction using the Quality of Life Inventory (Frisch et al., 1994) and the Satisfaction with Life Scale (Diener et al., 1985). Among college students, the agency component of hope, which refers to motivation and perseverance in pursuing academic goals, along with pessimism, predicted life satisfaction. This aligns with the idea that younger students may still be developing skills to reframe negative experiences and foster positive expectations (Bryant and Cvengros, 2004). In adult samples, both agency and optimism predicted life satisfaction, with agency consistently identified as a predictor in both groups.

Several studies (e.g., Gallagher and Lopez, 2009; Rand et al., 2020) have shown that hope and optimism predict changes in subjective wellbeing, with hope being a stronger predictor of life satisfaction and positive affect, while optimism is more associated with negative wellbeing. Hope, grounded in the belief of achieving controllable goals, fosters emotional wellbeing and contributes to academic satisfaction. Rand et al. (2020) argue that hope, driven by meaningful goals, is a stronger predictor of life satisfaction than optimism, which reflects general positive expectations.

Academic hope contributes to positive outcomes that enhance student life satisfaction. Azadianbojnordi et al. (2022) found a positive relationship between academic hope and engagement, mediated by academic buoyancy. Hopeful students are more likely to invest effort toward academic success, regulate challenges, and experience greater life satisfaction. Similarly, Lewis et al. (2011) showed that cognitive engagement, influenced by hope, predicted changes in students’ life satisfaction, with hopeful students who believed their academic environment would help achieve their goals reporting higher satisfaction over time.

## Methods

### Participants

Participants in this study are college students enrolled in higher education institutions in the Philippines during the 2023–2024 academic year. For the qualitative portion, stratified sampling was used, with written interview questionnaires distributed at a college within the researcher’s affiliated institution. Quota and convenience sampling were applied for participant recruitment, and study information was shared via the researcher’s Facebook. Data collection occurred through online survey forms (Google Forms), with all questionnaires converted to an online format. First-year students were excluded due to insufficient exposure to the school environment, which could influence satisfaction judgments.

#### Sample size

The sample size for the study was determined across two phases. In Phase 1, following Tabachnick et al. (2013) recommendation for a minimum of *n* = 300 for Exploratory Factor Analysis (EFA), the quantitative portion adhered to this guideline with a sample size of *n* = 300. In Phase 2, for the validation of CSLSS, an *a priori* power analysis conducted using G*Power estimated the required sample size to range from *n* = 96 to *n* = 138, with a significance level of 0.05 and power between 85 and 90%. This sample size was used for the correlation analyses.

### Research instruments

#### Research-made survey interview questionnaire

The participants were asked to respond to one open-ended question in writing: “In your experience as a student, what makes your life as a student satisfying? Give two things and explain.” They were instructed to write their two responses separately on different pieces of paper. The question was translated into Filipino to ensure that it was easily understood by the students. Filipino language experts validated the translated version of the question.

#### College student life satisfaction scale (CSLSS; Masagca, 2024)

The College Student Life Satisfaction Scale (CSLSS) assesses students’ satisfaction with their overall higher education experience, including academic programs and subjective interactions with peers and professors. Satisfaction is based on what students value most, with those reporting high life satisfaction perceiving their experiences in these areas as positive. In higher education, peers, teachers, and the institutional climate play key roles in fulfilling students’ psychological needs—competence, autonomy, and relatedness—leading to greater satisfaction through positive interactions. The CSLSS includes five dimensions: Peers, Faculty, School Environment, School-Life Balance, and Academic Performance. Sample items for each dimension are: “My professors are considerate of our circumstances” (Faculty); “I have a good relationship with my friends” (Peers); “My school has enough learning facilities for me to become a competent student” (School Environment); “I make my parents proud by achieving high grades in my subjects” (Academic Performance); and “I rarely find time for other things because of my coursework” (School-Life Balance). Responses are based on a Likert scale (1 = Strongly Disagree to 6 = Strongly Agree), with subscale scores calculated by averaging the items within each dimension. The overall satisfaction score is the sum of the five subscale scores.

#### Multidimensional student life satisfaction scale

The Multidimensional Student Life Satisfaction Scale (MSLSS; Huebner and Gilman, 2002) measures student life satisfaction across five dimensions: family, school, friends, self, and living environment. Sample items include: “My friends are nice to me” (Friends); “I am fun to be around” (Self); “I feel bad at school” (School); “I like spending time with my parents” (Family); and “There are lots of fun things to do where I live” (Living Environment). Responses are based on a six-point Likert scale (1 = Strongly Disagree to 6 = Strongly Agree). The scale’s reliability has been confirmed in four American and Canadian samples, with an overall alpha coefficient ranging from 0.90 to 0.92. The alphas for individual dimensions are: Family (0.79–0.85), Friends (0.81–0.85), School (0.83–0.85), Self (0.72–0.84), and Living Environment (0.79–0.83).

#### College satisfaction scale

The College Satisfaction Scale (CSS; Lodi et al., 2017) evaluates college student satisfaction across dimensions such as degree suitability, university services, peer relationships, study motivation, and career relevance. Sample items include: “After all, my degree course suits me” (Degree Suitability); “University employees are generally very helpful” (University Services); “I can perform many activities with my fellow students” (Peer Relationships); “I am very motivated to study” (Study Motivation); and “I feel that my studies will be useful for my future job” (Career Relevance). Responses are recorded on a five-point Likert scale (1 = Not at all to 5 = Extremely). Reliability indices (McDonald’s Omega) for the dimensions range from 0.84 to 0.92, as shown among psychology students and mixed samples.

#### Satisfaction with life scale

The Satisfaction with Life Scale (SWLS; Diener et al., 1985) measures global life satisfaction with five items, such as: “In most ways, my life is close to ideal,” “I am satisfied with my life,” and “If I could live my life over, I would change almost nothing.” Respondents use a seven-point Likert scale (1 = Strongly Disagree to 7 = Strongly Agree). Pavot and Diener (1993) reported a coefficient alpha range of 0.79 to 0.89 across six studies.

#### Academic hope scale

The Academic Hope Scale (AHS; Shorey and Snyder, 2004) measures academic hope with nine items rated on an eight-point Likert scale (1 = “definitely false” to 8 = “definitely true”). It includes two components: pathways and agency. Sample items for pathways are: “I actively pursue my educational goals,” “I take classes that are challenging to me,” and “Thinking about pursuing my goals in school fills me with energy.” Items for agency include: “I can think of many ways to make good grades,” “I can think of specific ways to do well in my classes,” and “The educational goals I have set for myself are clear and well defined.”

### Research design

The study used a mixed-methods approach. In Phase 1, qualitative data were collected to explore students’ perspectives on what makes student life satisfying. Rather than personal interviews, responses were gathered through a questionnaire with open-ended questions. This approach is suitable for test development, as it starts with conceptualizing the construct of student life satisfaction. Emerging themes from students’ responses formed the dimensions of this construct, and preliminary items were generated based on these themes. The quantitative phase focused on validating the factor structure of the student life satisfaction measurement model through factor analysis. Reliability and validity were established using statistical analyses, including correlational analysis and linear regression.

### Data analysis

Thematic analysis (Braun and Clarke, 2012) was conducted to identify common themes in students’ responses regarding factors related to their student life satisfaction. Exploratory Factor Analysis (EFA) was used to determine the factor structure of the CSLSS, as it is well-suited for theory development (Tabachnick et al., 2013). The study assumed that student life satisfaction results from the satisfaction of specific school-related aspects, with various factors influencing the latent variable of student life satisfaction.

Factor extraction was guided by the Maximum Likelihood (ML) method, which is optimal for data that are approximately normally distributed, as the items were expected to demonstrate relatively normal distributions (West et al., 1995; Fabrigar et al., 1999). ML allowed for the computation of goodness-of-fit indexes, statistical significance testing of factor loadings and correlations among factors, and calculation of confidence intervals. The Oblimin rotation method, which assumes some correlation between factors, was used, as human behavior is rarely neatly divided into independent units (Costello and Osborne, 2019).

For factor retention, only factors with eigenvalues >1 were kept, following the Kaiser-Guttman criterion (Zwick and Velicer, 1986). This rule ensures that retained factors have greater explanatory power than individual variables with a maximum eigenvalue of 1.0. Criterion-related validity was established through Linear Regression Analysis, using the CSLSS as the predictor and the Satisfaction with Life Scale as the outcome variable. Concurrent validity was assessed by correlating the CSLSS with the School domain of the Multidimensional School Life Satisfaction Scale (MSLSS) and the overall score on the College Satisfaction Scale (CSS). Discriminant validity was demonstrated through an independent *t*-test comparing the top 25% of high scorers and the bottom 25% of low scorers on the CSLSS.

### Procedure

Upon approval from the Manila Central University Institutional Review Board (MCU ERB; protocol no: 2024-073), the researcher began data collection for Phase 1. In this phase, students completed written interview questionnaires, and their responses were analyzed through thematic analysis. The emerging themes were used to generate preliminary items for the CSLSS. A pilot version of the CSLSS was then administered to students until the target quota sample size was reached. Exploratory Factor Analysis was conducted to empirically determine the factor structure of the CSLSS.

In Phase 2, the measurements, including the first version of the CSLSS, were converted into online survey forms and distributed to students. Once the target sample size was achieved, the online survey was closed, and the collected data were analyzed. After the data analysis, all anonymized research data were posted to the Open Science Framework, and all participants received an email containing a copy of the CSLSS.

### Ethical considerations

#### Participation

The selected participants had the freedom to refuse participation in the study. At any point during the study, they were free to withdraw without any penalty or loss of benefits. As a result, the data from participants who chose to withdraw were excluded from the study. The study posed minimal risks, and in cases where a participant felt discomfort, they were encouraged to withdraw.

#### Confidentiality

In the capacity of the researcher, the data were kept confidential. Codes were assigned to each participant to protect their anonymity and ensure privacy. No identifying information was included in any reports or publications related to the study. If there were any changes in the approved research procedures, the participants were informed, and they had the freedom to withdraw their data.

#### Data management

For qualitative data collection, participants were not required to provide personal information such as their name, class section, or academic program. Any identifying information written by the participants in their responses was deleted. As in the quantitative phases, all information that could compromise respondent anonymity was removed. The data were stored in encrypted cloud storage, accessible only to the researcher.

The anonymized data were uploaded to an open science platform, the Open Science Framework, to promote scientific transparency. This allowed the research data to be available for review by other researchers in subjective wellbeing scholarship and for potential research reproducibility. The results were shared through research reports, conferences, articles, and publications. Upon completion of the study, participants could request a copy by emailing the researcher at ramonpaulomasagca@gmail.com.

## Results

### Qualitative data

The researcher ensured that the items on the scale accurately reflect the true learning experience of Filipino college students by first exploring the dimensions of student life satisfaction through a qualitative study design. The domains that students refer to when evaluating their satisfaction with their learning experience include Faculty, Peers, School Environment, Academic Performance, and School-life Balance.

#### Faculty

Faculty play a pivotal role in shaping student learning outcomes, with their competence significantly influencing students’ acquisition of knowledge and skills. Students believe that faculty should ensure no student is left behind, ensuring full comprehension before moving to the next topic. The social connection between faculty and students also serves as a critical source of learning satisfaction. Professors who demonstrate flexibility, such as adjusting deadlines or accepting late submissions, are highly valued by students. Moreover, in a higher education setting that emphasizes critical thinking, students appreciate professors who are open to disagreements, seeing it as a sign of mutual respect despite the faculty-student hierarchy. Many students also find it comforting to discuss personal matters with professors, often referring to them as “second parents.”

#### Peers

Students spend a significant amount of time on campus, where their main focus is learning and preparing for the future. College also provides opportunities to build friendships with peers who face similar academic challenges. These shared experiences help make the journey more manageable, with classmates offering support and forming genuine, meaningful relationships. This sense of camaraderie becomes a key aspect of student life. Participants often report seeking help from classmates when struggling to understand a lesson. Many find that peer explanations enhance their understanding of the material, highlighting the importance of collaborative learning and peer support, particularly during group activities.

#### School environment

The learning environment is greatly impacted by the institution students attend. Adequate facilities are essential for fostering a productive learning atmosphere that helps students develop their competence. Students expect their institution to prepare them for real-world challenges after graduation. They specifically call for a sufficient number of faculty members and recommend assigning professors to courses aligned with their areas of expertise. Additionally, students report higher satisfaction when the quality of education matches the tuition fees they pay, with this concern being more pronounced among those in private institutions due to the lack of tuition subsidies.

#### Academic performance

Students derive satisfaction from their academic achievements, viewing strong grades as a reflection of their hard work and capabilities. Academic success provides them with a sense of mastery and fulfillment. Additionally, students see academic performance as a way to reciprocate the support of their guardians, particularly parents, who finance their education. Achieving high marks brings contentment, as it allows them to make their parents proud.

#### School-life balance

While students dedicate substantial time to campus activities, they find fulfillment in pursuits outside of academics. Many report greater happiness when their schedules allow for rest or leisure, such as spending time with family and friends. However, some experience guilt when engaging in non-academic activities, indicating the high value they place on their education. This dynamic can lead to frustration and impact overall life satisfaction. Students prioritize maintaining a healthy balance between academic and personal lives, which they believe can be achieved through effective time management, a structured academic schedule, and extracurricular opportunities provided by the school.

### Preliminary items

The items were generated based on the student life satisfaction domains that emerged from the thematic analysis. These domains serve as the subscales of the College Student Life Satisfaction Scale (CSLSS). Table 1 presents the items categorized per domain. The normality of the item distributions was examined by inspecting their skewness and kurtosis values. According to Gravetter and Wallnau (2011) and George (2011), skewness values between −2 and +2 are considered acceptable and indicative of approximate normality. Similarly, West et al. (1995) recommend that kurtosis values within the range of −7 to +7 are suitable for factor analysis. These thresholds offer reasonable assurance that the distribution of item responses does not significantly violate the assumption of normality. Consequently, item CSLSS78 was excluded from the exploratory factor analysis, as its skewness exceeded the recommended cutoff. Refer to Table 2 for the skewness and kurtosis values of each item.

**Table 1:** Preliminary items of CSLSS.

Dimensions	Item code	Item
Faculty	CSLSS1	I can easily approach my professors.
	CSLSS2	I do not feel anxious when my professors enter the classroom.
	CSLSS3	Professors empathize well with our situation as students.
	CSLSS4	Aside from teaching us, our professors serve as our second parents.
	CSLSS5	My professors are considerate of our circumstances.
	CSLSS6	Most of my professors value our opinions as students.
	CSLSS7	Our professors are open to comments during discussions.
	CSLSS8	My professors respect my boundaries as a student.
	CSLSS9	Our professors integrate real-life applications into their lessons.
	CSLSS10N	My professors are unreasonably strict.
	CSLSS11	Professors put in great effort to teach their subjects very well.
	CSLSS12	Professors ensure that all students understand the lesson before proceeding to the next.
	CSLSS13	I would say that I learn a lot from my professors.
	CSLSS14	Professors teach the courses they are experts in.
	CSLSS15	I can say that my school has enough faculty members to teach all the academic courses.
Peers	CSLSS16	I have a good relationship with my friends.
	CSLSS17	I have classmates whom I consider friends at school.
	CSLSS18	I enjoy working with my friends during group activities.
	CSLSS19	I struggle to find a group whenever my professor assigns a group activity.
	CSLSS20	My friends make my student life more bearable.
	CSLSS21	Whenever I have a problem, I can easily share it with my friends.
	CSLSS22	I am ready to help my peers when they are in need.
	CSLSS23	My friends at school genuinely care about me.
	CSLSS24	My friends at school value my opinion.
	CSLSS25	I would say that my friends share similar interests with me.
	CSLSS26	When needed to decide, I rely on my friends’ decisions.
	CSLSS27	My friends and I often reach the same decisions.
	CSLSS28	I can easily agree with my friends on certain things.
	CSLSS29	I can freely express myself when I am with my friends.
	CSLSS30	My friends help me uncover new perspectives on life.
	CSLSS31	My peers respect my personal decisions and choices in life.
	CSLSS32	I have friends I can count on whenever I do not understand a lesson.
	CSLSS33	Studying with friends is helpful for me.
	CSLSS34	I am more motivated to attend classes when I am with my friends.
	CSLSS35	My friends and I engage in friendly competition to see who can achieve the highest grade.
	CSLSS36	Having supportive friends drives me to excel in my studies.
	CSLSS37	I feel more capable when working with my peers on school activities.
	CSLSS38	I would say that I contribute meaningfully to group tasks.
School environment	CSLSS39	I feel a sense of belonging in our school community.
	CSLSS40N	I feel left behind at my school.
	CSLSS41	The school staff are friendly and approachable.
	CSLSS42	The school promotes inclusivity and encourages diversity within our community.
	CSLSS43	I feel a strong sense of community and belonging at my school.
	CSLSS44	I can freely express myself at school.
	CSLSS45	The school has nonacademic activities that help students grow.
	CSLSS46	I feel safe on campus.
	CSLSS47	My school has areas where students can spend their free time.
	CSLSS48	I believe the quality of education I receive is commensurate with the tuition I pay.
	CSLSS49	There are extracurricular activities or events for students from time to time.
	CSLSS50	Our school environment is conducive to learning.
	CSLSS51	I can say that my school has enough learning facilities for me to become a competent student.
	CSLSS52	The learning facilities are accessible to all students.
	CSLSS53N	Some of the school facilities are underutilized by students.
	CSLSS54	There are places dedicated to being “study spots.”
	CSLSS92	Overall, the school is making me equipped for my future career.
Academic performance	CSLSS91	I recognize that there is more to be gained beyond just good grades.
	CSLSS55	I make those around me proud by achieving high grades.
	CSLSS56	I feel more accepted by others when I get good grades.
	CSLSS57	I feel that my academic performance is acknowledged and appreciated by others.
	CSLSS58	I excel in my studies despite the challenges I face.
	CSLSS59	I make sure that I learn a lot from my studies.
	CSLSS61	I can easily adjust to the workloads given by my professors.
	CSLSS62	I believe that I will successfully finish my studies.
	CSLSS63	I am able to share my ideas in class without feeling too anxious.
	CSLSS64	I feel joy whenever I do well in class.
	CSLSS65	My grades do matter to me.
	CSLSS66	I tend to get good grades in most of my subjects.
	CSLSS67	I feel good whenever I get good grades.
	CSLSS68	I make my parents proud by achieving high grades in my subjects.
	CSLSS69	My grades are very important to me as they reflect my hard work.
	CSLSS70	I like to work on my activities as the deadline approaches.
	CSLSS71	I enjoy figuring out lessons on my own.
	CSLSS72	My efforts pay off whenever I get good grades.
School-life balance	CSLSS73	I still find time to study despite my responsibilities, such as family, work, and other commitments.
	CSLSS74	I am still able to maintain my relationships outside of school.
	CSLSS75	I am still able to find time to rest despite having academic responsibilities.
	CSLSS76	I can rest without being anxious about my pending schoolwork.
	CSLSS77	I do not find it difficult to set aside leisure time for myself.
	CSLSS78	I feel relieved whenever I am done with all of my academic responsibilities.
	CSLSS79	I have a good class schedule that enables me to do other things.
	CSLSS80	I can say that I manage my time effectively.
	CSLSS81N	I feel guilty resting because of my academic responsibilities.
	CSLSS82N	I constantly find myself juggling my responsibilities in school and my personal life.
	CSLSS84N	I tend to adjust my sleeping time just to have time to enjoy.
	CSLSS85N	I find myself having little free time because of school tasks.
	CSLSS86N	I rarely find time for other things because of my coursework.
	CSLSS87	I am able to use my spare time outside of school for general self-improvement.
	CSLSS88	I can easily be engaged in my studies because I have the things I need.

**Table 2:** Skewness and Kurtosis of CSLSS preliminary items.

Items	Skewness	Kurtosis
CSLSS1	−0.45	0.10
CSLSS2	−0.48	−0.05
CSLSS3	−0.74	1.39
CSLSS4	−0.22	−0.18
CSLSS5	−0.63	0.96
CSLSS6	−0.41	0.45
CSLSS7	−0.60	0.53
CSLSS8	−1.28	3.24
CSLSS9	−0.71	1.44
CSLSS10N	0.35	−0.12
CSLSS11	−0.49	0.24
CSLSS12	−0.63	0.43
CSLSS13	−0.81	1.84
CSLSS14	−0.84	1.15
CSLSS15	−0.20	−0.79
CSLSS16	−0.96	1.58
CSLSS17	−1.57	3.70
CSLSS18	−0.87	1.16
CSLSS19	0.53	−0.59
CSLSS20	−1.19	2.28
CSLSS21	−0.25	−0.64
CSLSS22	−0.69	0.56
CSLSS23	−0.93	1.43
CSLSS24	−0.82	1.37
CSLSS25	−0.84	1.70
CSLSS26	−0.12	−0.56
CSLSS27	−0.45	0.96
CSLSS28	−0.63	0.27
CSLSS29	−1.08	1.23
CSLSS30	−0.78	0.76
CSLSS31	−0.78	1.36
CSLSS32	−1.29	1.70
CSLSS33	−0.72	−0.01
CSLSS34	−0.99	1.02
CSLSS35	0.24	−0.83
CSLSS36	−1.02	1.62
CSLSS37	−0.68	1.05
CSLSS38	−0.77	0.68
CSLSS39	−0.64	0.93
CSLSS40N	0.44	−0.63
CSLSS41	−0.59	0.80
CSLSS42	−0.75	0.83
CSLSS43	−0.61	0.82
CSLSS44	−0.54	0.12
CSLSS45	−0.67	0.21
CSLSS46	−0.87	1.44
CSLSS47	−1.07	1.05
CSLSS48	−0.45	−0.03
CSLSS49	−0.81	1.01
CSLSS50	−0.53	1.22
CSLSS51	−0.57	0.50
CSLSS52	−0.71	0.46
CSLSS53N	−0.35	0.39
CSLSS54	−1.17	1.84
CSLSS55	−0.73	0.94
CSLSS56	−0.57	−0.14
CSLSS57	−0.47	0.28
CSLSS58	−0.59	0.90
CSLSS59	−0.67	1.72
CSLSS61	−0.54	0.68
CSLSS62	−1.20	1.21
CSLSS63	−0.30	−0.63
CSLSS64	−1.22	2.13
CSLSS65	−1.53	2.81
CSLSS66	−0.59	0.70
CSLSS67	−1.51	3.05
CSLSS68	−1.16	1.90
CSLSS69	−1.12	1.72
CSLSS70	−0.82	0.22
CSLSS71	−0.58	0.25
CSLSS72	−1.30	2.40
CSLSS73	−1.02	1.89
CSLSS74	−1.20	2.54
CSLSS75	−0.81	0.78
CSLSS76	0.23	−0.93
CSLSS77	−0.45	0.22
CSLSS78	−2.16	6.59
CSLSS79	−0.62	−0.31
CSLSS80	−0.51	−0.02
CSLSS81N	−0.63	−0.26
CSLSS82N	−0.38	0.09
CSLSS84N	−0.89	0.44
CSLSS85N	−0.36	−0.19
CSLSS86N	−0.19	−0.48
CSLSS87	−0.56	0.47
CSLSS88	−0.63	0.52
CSLSS92	−0.82	1.75

### Factor structure of CSLSS

**Figure 1:**
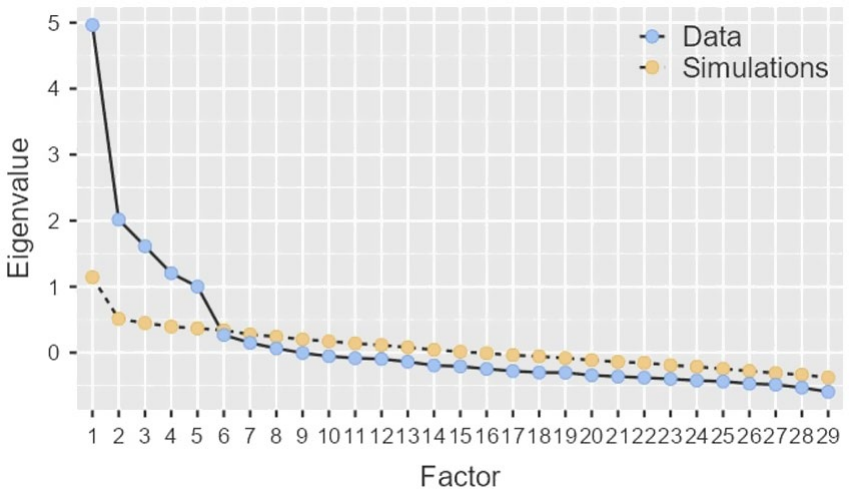
Scree plot.

The researcher applied Exploratory Factor Analysis to determine whether the domains of CSLSS are supported empirically. The Maximum Likelihood method was selected for extraction, combined with Oblimin rotation. Factors with eigenvalues >1 were retained, following the scree plot and parallel analysis also supporting the decision to keep five factors (see Figure 1 below).

The cumulative percentage of variance is 41.65%, which meets the threshold commonly deemed acceptable in social sciences. Bartlett’s Test of Sphericity yielded a statistically significant result (*p* < 0.00001), indicating that the correlation matrix is not an identity matrix and that significant relationships exist among the variables. Furthermore, all items of the CSLSS demonstrated Kaiser-Meyer-Olkin (KMO) Measure of Sampling Adequacy values exceeding 0.70, signifying a high proportion of shared variance among the variables. While Bartlett’s Test confirms the presence of sufficient correlations to justify factor analysis, the KMO Measure evaluates the adequacy of these relationships for uncovering underlying factors (see Tables 3–5).

**Table 3:** Factor statistics of CSLSS.

Dimensions	SS loadings	% of variance	Cumulative %	Eigenvalue
Peers	2.69	9.28	9.28	4.96
Faculty	2.65	9.13	18.41	2.02
School environment	2.34	8.05	26.47	1.61
Academic performance	2.22	7.66	34.13	1.20
School-life balance	2.18	7.52	41.65	1.00

**Table 4:** Bartlett’s test of sphericity.

*χ^2^*	*df*	*p*
3,726	406	<0.00001

**Table 5:** KMO measure of sample adequacy.

Variables	KMO MSA
Overall	0.83
CSLSS20	0.85
CSLSS32	0.84
CSLSS23	0.86
CSLSS16	0.86
CSLSS18	0.89
CSLSS5	0.86
CSLSS3	0.84
CSLSS6	0.89
CSLSS12	0.85
CSLSS1	0.83
CSLSS4	0.89
CSLSS51	0.85
CSLSS92	0.89
CSLSS48	0.86
CSLSS54	0.78
CSLSS47	0.8
CSLSS15	0.76
CSLSS86N	0.7
CSLSS81N	0.78
CSLSS75	0.8
CSLSS76	0.76
CSLSS85N	0.71
CSLSS77	0.83
CSLSS79	0.82
CSLSS68	0.83
CSLSS55	0.84
CSLSS72	0.84
CSLSS66	0.88
CSLSS67	0.79

#### Demographics

The preliminary items of the CSLSS were completed by 406 Filipino college students during the first semester of AY 2024–2025 via an online survey. Most respondents attended private institutions (67.0%), followed by public schools (27.6%), with 5.4% undisclosed. The majority were full-time students (87.9%), while 12.1% were working students. By year level, 44.6% were second-year, 35.5% third-year, and 20.0% fourth-year students. Females comprised 70.0% of the sample, while males accounted for 30.0%. For gender identity, 60.1% identified as feminine, 24.9% as masculine, and 15.0% as LGBTQIA+. Ages ranged from 18 to 24 years, with a mean of 20.25 years (SD = 1.53) (see Table 6).

**Table 6:** Demographic profile of respondents.

Demographics	*n*	% of total	Cumulative %
Higher education institution			
Did not disclose	22	5.42%	5.42%
Private college/university	272	67.00%	72.41%
Public college/university	112	27.59%	100.00%
Status			
Full-time student	357	87.93%	87.93%
Working student	49	12.07%	100.00%
Present year level			
2nd year	181	44.58%	44.58%
3rd year	144	35.47%	80.05%
4th year	81	19.95%	100.00%
Sex			
Female	284	69.95%	69.95%
Male	122	30.05%	100.00%
Gender			
Feminine	244	60.10%	60.10%
LGBTQIA+	61	15.03%	75.12%
Masculine	101	24.88%	100.00%

Demographics	*n*	Mean	SD
Age	406	20.25	1.53

### Item removal process

The researcher employed a systematic approach to exploratory factor analysis. Items with cross-loadings >0.3 on unrelated factors were removed, starting with the factor with the highest explained variance. After purifying the dominant factor, they addressed cross-loading items on subsequent factors with lower explained variance. If an item reappeared as a cross-loading on the primary factor, it was iteratively removed until no cross-loadings above 0.3 remained on the strongest factor. Items with factor loadings below 0.35 were then eliminated, beginning with the most stable factor. The 0.35 cut-off aligns with Hair et al. (2006) recommendation for samples of *n* > 350. This iterative process adheres to best practices in exploratory factor analysis. See Table 7 for the factor loadings of the dimensions.

**Table 7:** Factor loadings of CSLSS subscales.

Items	Dimensions					Uniqueness
	Peers	Faculty	School environment	Academic performance	School-life balance	
CSLSS20	**0.750**	−0.010	−0.040	0.000	0.010	0.451
CSLSS32	**0.730**	−0.020	−0.060	−0.060	0.000	0.509
CSLSS23	**0.720**	0.050	0.060	0.080	−0.020	0.399
CSLSS16	**0.720**	0.010	0.010	0.060	−0.020	0.447
CSLSS18	**0.620**	0.060	0.060	−0.050	0.100	0.577
CSLSS5	**0.040**	0.760	−0.040	0.060	0.050	0.379
CSLSS3	−0.050	**0.710**	−0.050	0.070	0.020	0.507
CSLSS6	0.010	**0.600**	0.200	0.020	−0.030	0.506
CSLSS12	0.070	**0.550**	0.080	−0.070	−0.030	0.659
CSLSS1	0.060	**0.550**	0.050	−0.060	0.110	0.645
CSLSS4	0.080	**0.520**	0.120	0.060	−0.130	0.625
CSLSS51	−0.040	0.130	**0.630**	−0.070	0.040	0.546
CSLSS92	0.110	0.130	**0.600**	0.020	−0.050	0.533
CSLSS48	−0.060	0.050	**0.560**	0.030	0.000	0.662
CSLSS54	0.020	−0.090	**0.530**	0.150	0.000	0.689
CSLSS47	0.160	−0.130	**0.530**	0.030	0.060	0.695
CSLSS15	−0.090	0.130	**0.530**	−0.060	0.010	0.676
CSLSS68	0.000	0.020	0.000	**0.760**	0.000	0.411
CSLSS55	0.030	0.090	−0.080	**0.710**	0.010	0.471
CSLSS72	0.090	0.000	0.050	**0.580**	−0.090	0.609
CSLSS66	−0.020	0.060	0.010	**0.500**	0.140	0.704
CSLSS67	0.060	−0.040	0.110	**0.500**	−0.090	0.698
CSLSS86N	−0.070	−0.030	0.140	0.000	**−0.690**	0.516
CSLSS81N	−0.040	−0.020	0.010	0.160	**−0.590**	0.632
CSLSS75	0.030	−0.150	0.200	0.250	**0.560**	0.560
CSLSS76	−0.070	0.070	0.160	−0.070	**0.520**	0.672
CSLSS85N	0.040	−0.090	0.180	0.030	**−0.510**	0.720
CSLSS77	0.000	0.030	0.160	−0.040	**0.490**	0.706
CSLSS79	−0.060	0.130	0.110	0.210	**0.400**	0.718

The numbers in bold indicate the factor loadings of the items under each component.

### Validation of CSLSS

#### Demographics

The demographic profile of respondents in the CSLSS validation study is as follows: Most were enrolled in private institutions (79.51%, *n* = 260), with 20.49% (*n* = 67) from public schools. The majority were full-time students (88.38%, *n* = 289), while 11.62% (*n* = 38) were working students. Second-year students comprised the largest group (62.69%, *n* = 205), followed by third-year (21.41%, *n* = 70) and fourth-year students (15.90%, *n* = 52). In terms of sex, 60.86% (*n* = 199) identified as female and 39.14% (*n* = 128) as male. Regarding gender identity, 54.43% (*n* = 178) identified as feminine, 34.86% (*n* = 114) as masculine, and 10.70% (*n* = 35) as LGBTQIA+. The average age was 20.36 years (SD = 1.51), with a total sample of 327 (see Table 8 for details).

**Table 8:** Demographic profile of the respondents.

Demographics	*n*	% of total	Cumulative %
Higher education institution			
Private college/university	260	79.51%	79.51%
Public college/university	67	20.49%	100.00%
Status			
Full-time student	289	88.38%	88.38%
Working student	38	11.62%	100.00%
Present year level			
2nd year	205	62.69%	62.69%
4th year	52	15.90%	78.59%
3rd year	70	21.41%	100.00%
Sex			
Female	199	60.86%	60.86%
Male	128	39.14%	100.00%
Gender			
Feminine	178	54.43%	54.43%
Masculine	114	34.86%	89.30%
LGBTQIA+	35	10.70%	100.00%

Demographics	*n*	M	SD
Age	327	20.36	1.51

#### Concurrent validity

The purpose of assessing the concurrent validity of the CSLSS is to confirm that it effectively measures student life satisfaction among college students. While this might seem redundant, the CSLSS introduces a new dimension—school-life balance—that distinguishes it from other existing measures of student life satisfaction, such as the CSS and MSLSS. Table 3 presents the correlations between the CSLSS and the CSS and MSLSS. CSLSS Overall is positively associated with CSS Overall, *r* (362) = 0.68, *p* < 0.001, and MSLSS Overall, *r* (362) = 0.61, *p* < 0.001.

In terms of the subscales of CSLSS, it is also supported that they are related to the concurrent scales. Specifically, CSLSS Peers is positively correlated with CSS Overall, *r* (362) = 0.48, *p* < 0.001, and MSLSS Overall, *r* (362) = 0.44, *p* < 0.001. CSLSS Faculty is positively correlated with CSS Overall, *r* (362) = 0.51, *p* < 0.001, and MSLSS Overall, *r* (362) = 0.46, *p* < 0.001. CSLSS School Environment is positively correlated with CSS Overall, *r* (362) = 0.57, *p* < 0.001, and MSLSS Overall, *r* (362) = 0.50, *p* < 0.001. The school-life balance subscale of the CSLSS also shows a positive correlation, though weaker, with CSS Overall, *r* (362) = 0.17, *p* < 0.01, and MSLSS Overall, *r* (362) = 0.23, *p* < 0.001. Additionally, CSLSS Academic Performance is positively correlated with CSS Overall, *r* (362) = 0.51, *p* < 0.001, and MSLSS Overall, *r* (362) = 0.40, *p* < 0.001 (see Table 9).

**Table 9:** Concurrent validity of CSLSS.

CSLSS	Concurrent scales	
	CSS overall	MSLSS overall
Overall	0.68***	0.61***
Peers	0.48***	0.44***
Faculty	0.51***	0.46***
School environment	0.57***	0.50***
School-life balance	0.17**	0.23***
Academic performance	0.51***	0.40***

*p < 0.05, **p < 0.01, ***p < 0.001.

### Criterion-related validity

#### CSLSS and SWLS

A simple linear regression analysis was performed to examine the predictive relationship between student life satisfaction and general life satisfaction. The regression model was statistically significant, *R*^2^ = 20, *F*_(1,362)_ = 80.47, *p* < 0.0001, and explained 20% of the variance in SWLS. The findings indicate that student life satisfaction is a significant predictor of global life satisfaction, *B* = 1.08, (362) = 8.97, *p* < 0.0001, 95% [0.84, 1.31] (see Table 10).

**Table 10:** CSLSS positively predicts SWLS.

Predictor	*B*	Standard error	95% confidence interval		*t*	*p*
			Lower	Upper		
Intercept	−2.28	2.66	−7.51	2.96	−0.85	0.39329
CSLSS overall	1.08	0.12	0.84	1.31	8.97	< 0.0001
R^2^ (proportion of variability accounted for by the predictor)	0.198 or 20%					

#### Academic hope and CSLSS

Academic hope has two dimensions: pathways and agency. To test if student life satisfaction predicted by Pathways and Agency, two single linear regressions were conducted. Pathways is a positive predictor of CSLSS, *B* = 0.25, (362) = 10.81, *p* < 0.0001, 95% [0.21, 0.30]. 26% variance in CSLSS is accounted for by the Pathway, *R*^2^ = 0.27, *F*_(1,362)_ = 116.92, *p* < 0.0001 (see Table 11).

**Table 11:** Pathways positively predicts CSLSS.

Predictor	*B*	Standard error	95% confidence interval		*t*	*p*
			Lower	Upper		
Intercept	15.94	0.57	14.81	17.06	27.90	< 0.0001
Pathways	0.25	0.02	0.21	0.31	10.81	< 0.0001
R^2^ (proportion of variability accounted for by the predictor)	0.266 or 27%					

The Agency dimension exhibits a comparable trend. Agency is a positive predictor of student life satisfaction, *B* = 0.21, (362) = 11.46, *p* < 0.0001, 95% [0.18, 0.25]. Additionally, Agency explains 26% of the variance in student life satisfaction, *R*^2^ = 0.29, *F*_(1,362)_ = 131.29, *p* < 0.0001 (see Table 12).

**Table 12:** Agency positively predicts CSLSS.

Predictor	*B*	Standard error	95% confidence interval		*t*	*p*
			Lower	Upper		
Intercept	15.64	0.57	14.52	16.76	27.57	< 0.0001
Agency	0.21	0.02	0.18	0.25	11.46	< 0.0001
R^2^ (proportion of variability accounted for by the predictor)	0.288 or 29%					

### Reliability

The five CSLSS dimensions exhibit acceptable inter-item consistency, with an overall Cronbach’s alpha of 0.87, indicating good item consistency. The subscales show the following results: Peers (0.85), Faculty (0.87), and School Environment (0.78), all demonstrating good to acceptable reliability. The School-life Balance subscale has a lower alpha of 0.67, while the Academic Performance subscale shows a Cronbach’s alpha of 0.76, indicating acceptable consistency (see Table 13).

**Table 13:** Cronbach’s alpha: CSLSS and its dimensions.

CSLSS	Cronbach’s *α*
Overall	0.87
Peers	0.85
Faculty	0.87
School environment	0.78
School-life balance	0.67
Academic performance	0.76

### Discriminant validity

Discriminant validity of the CSLSS was confirmed by comparing the lowest 25% and highest 25% of scores using Student’s and Welch’s *t*-tests. Student’s *t*-test was applied to the Faculty, School Environment, and School-life Balance dimensions, while Welch’s *t*-test was used for Overall, Peers, and Academic Performance due to significant variance differences. All *t*-tests yielded significant results, indicating substantial differences between the groups, with large effect sizes for all comparisons.

Welch’s *t*-test revealed a significant difference for the Overall scale, *t*(109.95) = 29.65, *p* < 0.00001, Cohen’s *d* = 5.10. Significant differences were also observed for the Peers dimension, *t*(77.94) = 9.28, *p* < 0.00001, Cohen’s *d* = 1.66, and the Academic Performance dimension, *t*(81.83) = 11.16, *p* < 0.00001, Cohen’s *d* = 1.99. Similarly, Student’s *t*-test showed significant differences for the Faculty dimension, *t*(143.00) = 18.80, *p* < 0.00001, Cohen’s *d* = 3.19, the School Environment dimension, *t*(143.00) = 15.97, *p* < 0.00001, Cohen’s *d* = 2.71, and the School-life Balance dimension, *t*(143.00) = 7.96, *p* < 0.00001, Cohen’s *d* = 1.35 (see Table 14).

**Table 14:** CSLSS discriminant validity.

Scales	Type	Statistic	*df*	*p*	Mean difference	SE difference	Effect size
Overall	Welch’s	29.65	109.95	<0.00001	6.81	0.23	5.10
Peers	Welch’s	9.28	77.94	<0.00001	1.31	0.14	1.66
Faculty	Student’s	18.80	143.00	<0.00001	1.78	0.09	3.19
School environment	Student’s	15.97	143.00	<0.00001	1.53	0.10	2.71
School-life balance	Student’s	7.96	143.00	<0.00001	0.94	0.12	1.35
Academic performance	Welch’s	11.16	81.83	<0.00001	1.24	0.11	1.99

## Discussion

The development of the College Student Life Satisfaction Scale (CSLSS) was prompted by the lack of an instrument specifically designed to measure college students’ satisfaction with their learning experiences. Existing scales, such as the College Satisfaction Scale (CSS; Lodi et al., 2017) and the School domain of the Multidimensional Student Life Satisfaction Scale (MSLSS; Huebner and Gilman, 2002), present psychometric limitations. The CSS was developed for Italian college students. Its direct application to Filipino college students raises concerns regarding cultural validity. Meanwhile, the School domain of the MSLSS primarily focuses on students’ general perceptions and feelings about school, without identifying which specific dimensions of the school contribute to their satisfaction. It lacks second-order domains that would allow for a more nuanced assessment of satisfaction within the school setting. These concerns regarding validity and factor structure were explicitly addressed in the development of the CSLSS.

The researcher aimed to ensure that the CSLSS accurately reflects the lived experiences of Filipino students, particularly their learning experiences in higher education. To achieve this, the scale items were developed based on themes that emerged from a qualitative analysis of students’ written responses to an interview question exploring which specific aspects of their college life contributed to their overall life satisfaction as students. The thematic analysis yielded five core domains: Peers, Faculty, School Environment, School-Life Balance, and Academic Performance.

The qualitative data revealed the influence of Filipino cultural values on students’ academic satisfaction. One salient value is *utang na loob*, or “debt of gratitude,” which was frequently expressed by students. For many Filipino college students, good grades—representing strong academic performance—are not merely a sign of personal achievement, but also a way to honor their parents’ sacrifices. Academic success is perceived as a means of giving back, fulfilling an implicit social contract within the family.

This cultural pattern aligns with findings from Bernardo (2010), who reported that Filipino students’ academic performance is closely linked to parental expectations and involvement. The study found that higher parental academic expectations were positively associated with students’ academic outcomes. Similarly, Tan (2022) highlighted the psychological strain Filipino students experience as they navigate the tension between their personal goals and the expectations imposed by others, including parents and a competitive academic environment.

Family occupies a central role in collectivist societies such as the Philippines, where strong familial ties and obligations are deeply embedded in social life. Among Filipino college students, this collectivist orientation is reflected in their constant effort to balance academic responsibilities with family duties. Fuligni (2001) found that students from collectivist cultures, including Asian and Latin American backgrounds, reported a stronger sense of family obligation compared to their European counterparts. These findings underscore the need to recognize the cultural context in which student life satisfaction is shaped.

Accordingly, pedagogical approaches that support school-life balance are essential—not only to promote academic engagement but also to enable students to fulfill their familial responsibilities. Both domains are deeply intertwined in the college experience and contribute to students’ overall sense of fulfillment.

Within the Filipino educational context, there is a prevalent belief that teachers serve as the students’ “second parents.” In this role, educators are viewed as central figures in shaping effective pedagogy and nurturing positive student–teacher relationships anchored in academic care. This cultural reverence for teachers as authority figures fosters student engagement and helps bridge the gap between teacher-centered and learner-centered pedagogical approaches (Del Valle, 2022).

Expanding on this cultural perspective, Filipino teachers often interpret supportive school relationships through a familial lens. As Viernes and de Guzman (2005) noted, these connections are characterized by reciprocity and emotional nourishment, reinforcing a school climate grounded in care, respect, and mutual responsibility. This culturally resonant framework highlights the importance of cultivating educational environments that reflect the values and relational expectations of the Filipino learner.

Exploratory Factor Analysis was employed to validate the factor structure of the developed scale. After the judicious item removal process, there are 29 items distributed across five domains: Faculty (6 items), Peers (5 items), School Environment (6 items), School-Life Balance (7 items), and Academic Performance (5 items).

The scale also demonstrated internal consistency across its items—both overall and within each domain. The School-Life Balance domain, however, yielded a slightly lower alpha coefficient compared to the conventional threshold of 0.70. Nonetheless, Taber (2018) notes that there is limited empirical support for such a rigid cutoff for inter-item consistency. The author argues that excessively high alpha values may indicate that items are overly similar or even redundant. Moreover, a high alpha does not necessarily imply that a construct is unidimensional. Similarly, Athanasiou and Mavrikaki (2014) caution that an instrument may encounter validity issues if the alpha is exceptionally high. In the case of the School-Life Balance domain of the CSLSS, the obtained alpha of 0.67 can still be considered acceptable. This is particularly reasonable given the novelty of this domain, which likely emerged due to blurred boundaries between school and home life during the pandemic (Soares et al., 2022). In such cases, a lower alpha during the pilot phase is expected and justifiable. Another reason is that life outside school is multifaceted. Students may allocate their time to various activities beyond academic responsibilities, such as pursuing work, attending to family obligations, engaging in leisure, and other meaningful pursuits. In line with this, the School-Life Balance domain consists of items that reflect these interests and responsibilities beyond the academic context. Given this, students may have differing priorities and commitments outside school, which could lead to varied responses to the items within the School-Life Balance domain. Consequently, this variability may influence the value of Cronbach’s alpha, as greater response diversity can result in lower inter-item consistency. This pattern suggests that the School-Life Balance domain may encompass distinct facets (e.g., rest, personal time, family engagement) that could later be empirically validated as its second-level domains. Such a structure implies a potential multidimensional substructure within the domain, warranting further psychometric evaluation.

To strengthen the psychometric rigor of the School-Life Balance domain within the CSLSS, it is recommended that future studies conduct an Exploratory Factor Analysis (EFA) to examine the presence of underlying subdimensions (e.g., rest, family engagement, personal time). Should distinct facets emerge, a hierarchical Confirmatory Factor Analysis (CFA) is warranted to test a nested factor structure. Model fit indices such as the CFI, TLI, and RMSEA should be evaluated to determine whether the proposed structure offers a superior fit to the data. Reliability coefficients should also be reported at both the subdimension and domain levels to assess internal consistency. Based on these findings, the identified facets may be formally established as second-level domains or used to refine the conceptual framework of the School-Life Balance construct.

This study establishes the validity of the College Student Life Satisfaction Scale. The goal was to evaluate the CSLSS’s concurrent validity, which means checking if it accurately measures college students’ life satisfaction. Although this assessment may seem unnecessary, the CSLSS includes a new dimension - school-life balance - that sets it apart from other existing student life satisfaction measures, such as the CSS and MSLSS. The findings show the CSLSS is associated with these concurrent scales. Concurrent validity is a crucial psychometric property of the CSLSS, as it was designed to be a “better” alternative to existing student life satisfaction scales (Cronbach and Meehl, 1955), reflecting the experiences of Filipino college students.

The criterion-related validity of the CSLSS suggests that students’ self-reported satisfaction with their college experience is a strong predictor of their overall life satisfaction (Satisfaction with Life Scale). Given that young adults dedicate a significant portion of their time to college life, the quality of their learning environment can profoundly impact their broader sense of wellbeing. Key aspects of the collegiate experience—such as relationships with peers, instructors, and family members, the quality of instruction, campus safety, and academic performance (Huebner et al., 2014)—play a critical role in meeting students’ basic psychological needs: competence, relatedness, and autonomy. For example, students establish friendships during their time in college (relatedness). Ideally, they have the autonomy to choose the academic program they want to pursue, which increases their engagement (autonomy). As a result, they are motivated to strive for competence, which leads to better academic performance (competence). When these needs are fulfilled, it contributes to higher levels of overall life satisfaction.

In general, being hopeful tends to make people feel more satisfied with their lives (Bryant and Cvengros, 2004; Bailey et al., 2007; Gallagher and Lopez, 2009; Rand et al., 2020). In the context of college education, this study shows that student life satisfaction is also predicted by academic hope, particularly through its two components: pathways and agency. As students become more hopeful, they feel more in control when facing academic challenges (Azadianbojnordi et al., 2022). When students have personally meaningful goals upon which their hope is founded, they are more likely to feel satisfied with their lives (Rand et al., 2020). Specifically, when students believe in their ability to initiate and sustain actions toward their academic goals (agency), they are more likely to feel satisfied with their student life (CSLSS). College students also have the ability to reframe negative thoughts and develop positive expectations about their experiences (Bryant and Cvengros, 2004). Moreover, as students become cognitively engaged in their academic plans, they are more likely to feel satisfied with their lives (Lewis et al., 2011). Similarly, student life satisfaction increases as students become more strategic in achieving their academic goals—developing the ability to generate and follow through with effective plans (pathways). When students have specific strategies to achieve their academic goals, they experience greater emotional wellbeing. Additionally, as students approach the realization of their goals, their academic satisfaction (and, in turn, their student life satisfaction) tends to increase (Lewis et al., 2011). Thus, the idea that hope positively predicts life satisfaction extends to contexts such as the academic setting, particularly in college education.

## Implication and conclusion

Although existing scales measuring student life satisfaction are available, it is essential to critically evaluate their applicability and relevance to specific populations. Domain-specific assessments—especially those tailored for college students—must capture the unique dimensions that shape their evaluative judgments. To ensure content validity, instruments like the CSLSS should be grounded in students’ lived experiences, thereby providing an accurate reflection of the realities of their learning environment in higher education.

Moreover, the test development process in the social sciences, particularly in psychology, should incorporate a rigorous integration of both qualitative and quantitative methodologies. Qualitative approaches, such as thematic analysis, offer rich insights into students’ lived experiences and perspectives, serving as a foundation for item generation. In parallel, quantitative methods—such as psychometric evaluations—are essential for establishing the scale’s reliability and validity. This comprehensive, mixed-methods approach enhances the scale’s capacity to accurately capture student life satisfaction and provides a robust framework for understanding and promoting the wellbeing of college students.

The development of the CSLSS responds to the significant transformations in higher education in the Philippines brought about by the COVID-19 pandemic. The convergence of school and home environments during remote learning posed substantial challenges for students in managing academic responsibilities alongside personal life demands. In the post-pandemic context, students have become increasingly aware of the importance of maintaining a healthy school-life balance, recognizing it as a critical factor that influences their overall satisfaction with college life.

Objective assessments are well-documented in the literature and have become a hallmark of quality assurance in higher education. They serve a vital function in guiding policymakers in the design and implementation of programs aimed at improving academic outcomes and institutional effectiveness. In the Philippines, for example, the Second Congressional Commission on Education (EDCOM II) has been established to evaluate the education system and recommend evidence-based reforms. Its mandate is to address long-standing issues related to quality, equity, and accessibility in education, while ensuring alignment with the competencies required in the 21st century to support national development.

While objective assessments are essential for evaluating measurable educational outcomes, the inclusion of subjective assessments offers equally important insights into students’ lived experiences. The College Student Life Satisfaction Scale (CSLSS), for instance, provides a means to assess college students’ satisfaction across key dimensions of their educational environment, including academic programs, social interactions, and campus resources. By integrating both objective and subjective measures, policymakers and educational leaders can gain a more comprehensive understanding of student wellbeing and institutional effectiveness, enabling them to identify targeted areas for improvement and promote a more holistic approach to educational reform.

### Directions for future researchers

The respondents of this study are predominantly from private higher education institutions. Hence, there is a need for scholarly efforts to investigate whether the scale is valid for measuring student life satisfaction among students enrolled in both private and public institutions. Such a validation study—potentially through confirmatory factor analysis to test the scale’s latent structure across institutional types—would be valuable, especially given the limited empirical evidence in the Philippine context examining whether student life satisfaction differs between these institutional types. For instance, a study conducted among nursing students in Central Luzon compared the satisfaction levels of those enrolled in private and public higher education institutions. The findings revealed that students in private institutions reported higher satisfaction in areas such as in-class teaching, clinical instruction, program structure, departmental support and resources, and overall satisfaction (Lumanlan, 2013). This disparity may be attributed to the greater financial flexibility of private institutions in resource allocation, particularly in budgeting (Castano and Cabanda, 2007), in contrast to state and local institutions that continue to experience reductions in government funding (Adeyemo, 2015). Moreover, students in public institutions often face compounded challenges due to their socioeconomic status. This was particularly evident during the COVID-19 pandemic, when many public-school students experienced unstable internet connectivity—a factor identified as a predictor of student life satisfaction (Cleofas, 2023).

In the analysis conducted during the validation phase of the present study, a significant difference was observed among gender groups—straight men (*n* = 114), straight women (*n* = 178), and individuals identifying as LGBTQIA+ (*n* = 35)—in the Peers and Academic Performance dimensions, as well as in overall CSLSS scores. A one-way ANOVA revealed statistically significant group differences for Peers [Welch’s *F*_(2, 117.71)_ = 8.74, *p* < 0.001], Academic Performance [Welch’s *F*_(2, 96.51)_ = 12.40, *p* < 0.001], and overall CSLSS scores [Welch’s *F*_(2, 97.56)_ = 3.37, *p* = 0.038]. In the Peers domain, LGBTQIA+ students (M = 5.23, SD = 0.50) and straight women (M = 5.03, SD = 0.84) reported higher satisfaction compared to straight men (M = 4.77, SD = 0.79). Similarly, in the Academic Performance domain, LGBTQIA+ students (M = 5.05, SD = 0.58) and straight women (M = 5.03, SD = 0.63) reported higher satisfaction than straight men (M = 4.63, SD = 0.74). In terms of overall college student life satisfaction (CSLSS total score), LGBTQIA+ students (M = 22.70, SD = 2.26) and straight women (M = 22.13, SD = 2.53) also reported higher satisfaction compared to straight men (M = 21.58, SD = 2.57). It is important to note that the relatively small sample size of LGBTQIA+ respondents (*n* = 35) warrants caution when generalizing the results to the broader LGBTQIA+ student population.

These Supplementary materials suggest that gender norms may influence how students assign value to specific dimensions of student life satisfaction. For instance, men who adhere to traditional masculine norms may avoid seeking academic or social support, perceiving such behaviors as signs of weakness—an attitude aligned with conventional masculinity scripts (Singh-Pillay and Naidoo, 2020). In contrast, individuals with nonbinary gender identities often face discrimination and negative stereotyping, even within academic settings. As a response, LGBTQIA+ students may engage in compensatory behaviors—such as striving for academic excellence—to counteract stigma and establish social credibility (Kasa, 2024; Ma and Li, 2025). A similar pattern is observed among women, who, despite facing systemic barriers, often demonstrate higher levels of academic engagement and diligence than men, particularly in male-dominated fields such as STEM (Pilotti, 2021). This increased engagement is sometimes attributed to their anticipation of discrimination and endorsement of egalitarian gender-role orientations (Xu et al., 2024).

Given these Supplementary materials, it is recommended that future researchers explore the validity of the CSLSS across diverse gender identities. Such efforts would be especially meaningful if further evidence establishes that gender orientation plays a significant role in shaping students’ learning experiences within the Philippine context. Additionally, future studies may investigate which specific dimensions of the CSLSS most strongly contribute to student life satisfaction across different populations—such as those based on gender orientation or institutional type—using structural equation modeling or hierarchical regression analysis.

Overall, the CSLSS has demonstrated strong validity and reliability as a tool for assessing the multidimensional nature of life satisfaction among Filipino college students. The scale was developed through a rigorous process that included qualitative item generation grounded in students’ lived experiences, followed by exploratory factor analysis to establish its factorial structure. Each of the identified domains—Peers, Faculty, Academic Performance, School Environment, and School-Life Balance—showed acceptable to high internal consistency. These findings affirm both the content and structural validity of the instrument. While opportunities for further research remain—particularly in validating the scale across diverse populations—the CSLSS offers a robust foundation for future studies examining the outcomes associated with a satisfying student life.

## Data Availability

The datasets presented in this study can be found in online repositories. The names of the repository/repositories and accession number(s) can be found in the article/Supplementary material.
